# Robust neutralizing antibody response to the XBB.1.5 trivalent recombinant protein vaccine booster

**DOI:** 10.1038/s41392-024-01924-y

**Published:** 2024-08-16

**Authors:** Bing-Dong Zhan, Xue-Dong Song, Xin Yu, Guo-Jian Yang, Sheng Wan, Mai-Juan Ma

**Affiliations:** 1 Quzhou Center for Disease Control and Prevention, Quzhou, China; 2 State Key Laboratory of Pathogen and Biosecurity, Academy of Military Medical Sciences, Beijing, China; 3 Qujiang District Center for Disease Control and Prevention, Quzhou, China; 4 Department of Microbiological Laboratory Technology, School of Public Health, Cheeloo College of Medicine, Shandong University, Jinan, China

**Keywords:** Infectious diseases, Infectious diseases

**Dear Editor,**

The global prevalence of XBB subvariants, known for their significant immune evasion, has driven the development and adaptation of XBB vaccines. Monovalent XBB.1.5 mRNA vaccines have been developed and shown to provoke robust immune responses against XBB.1.5, EG.5.1, and BA.2.86 following vaccination.^1^ In June 2023, the XBB.1.5-recombinant COVID-19 trivalent (XBB.1.5 + BA.5+Delta) protein vaccine (trivalent XBB.1.5 vaccine) (WestVac Biopharma Co., Ltd., China) was approved for emergency use against XBB subvariants. Although preliminary data from the manufacturer suggest neutralization of several earlier Omicron subvariants and XBB.1.5, there is limited real-world evidence, and persisting immune imprinting may affect the induction of antibodies against new SARS-CoV-2 sublineages,^2^ such as JN.1, which has increased transmissibility and the ability to evade immunity.^3^ Additionally, subsequent XBB infection after BA.5/BF.7 breakthrough infection does not efficiently induce humoral immunity against JN.1,^4^ raising questions about the ability of the trivalent XBB.1.5 vaccine to provide adequate protection against this lineage. We measured the neutralizing antibody responses in 32 individuals who experienced a BA.5/BF.7 breakthrough infection and a subsequent XBB infection before and after receiving the trivalent XBB.1.5 vaccination. The detailed demographic information of the study participants and their vaccination and infection histories are described in the Supplementary Methods.

Neutralizing antibodies against D614G, Delta, BA.5, BF.7, XBB.1.5, EG.5.1, and JN.1 were assessed in serum samples from 32 individuals who had received the trivalent XBB.1.5 vaccine before and three weeks after vaccination using a pseudotyped virus-based neutralization assay (Supplementary methods). We found that before vaccination, 100% of the serum samples had detectable neutralizing antibody titers against the D614G, BA.5, BF.7, XBB.1.5, and EG.5.1 variants. However, neutralizing antibody titers against Delta and JN.1 were detected in 30 (93.8%) and 26 (81.3%) of the 32 serum samples, respectively (Fig. 1a, b). The neutralization of Delta, BA.5, BF.7, XBB.1.5, and EG.5.1 was less effective compared to D614G, with 7.7-, 4.7-, 3.0-, 9.7-, and 9.6-fold reductions in geometric mean titers (GMTs), respectively. JN.1 neutralization was the least efficient, exhibiting 3.4-, 3.4-, 10.8-, 7.0-, 4.2-, and 32.7-fold decreases in GMTs compared to EG.5.1, XBB.1.5, BF.7, BA.5, Delta, and D614G, respectively (Fig. 1b). Notably, no significant differences were observed between the neutralization of XBB.1.5 and EG.5.1 (Fig. 1b). Three weeks after vaccination, neutralizing antibody titers against the JN.1 variant were detected in all the serum samples, while neutralizing antibody titers against the other tested variants remained detectable in all the serum samples (Fig. 1a, c). There was an 8.7-fold increase in GMT against D614G compared to pre-vaccination levels, with a GMT of 23875 (95% confidence interval [CI] 16006-35613). A greater increase in GMTs was observed for Delta, BA.5, BF.7, XBB.1.5, EG.5.1, and JN.1, ranging from 19.0-fold to 24.9-fold after vaccination (Fig. 1a). No significant differences in neutralizing titers against BA.5 (GMT 14539, 95% CI 8401-25160) or BF.7 (GMT 19684, 95% CI 11927-32485) were noted after vaccination compared to D614G. The serum neutralization titers against XBB.1.5 and EG.5.1 were similar but significantly lower than those against D614G, BA.5, and BF.7 by 2.4-to-4.0-fold. Overall, neutralization titers against JN.1 were the lowest, with a GMT of 1648 (95% CI 1042-2605), representing 14.5-, 4.1-, 8.8-, 11.9-, 3.6-, and 3.7-fold reductions in GMTs compared to the titers against D614G, Delta, BA.5, BF.7, XBB.1.5, and EG.5.1, respectively (Fig. 1c).

**Fig. 1:**
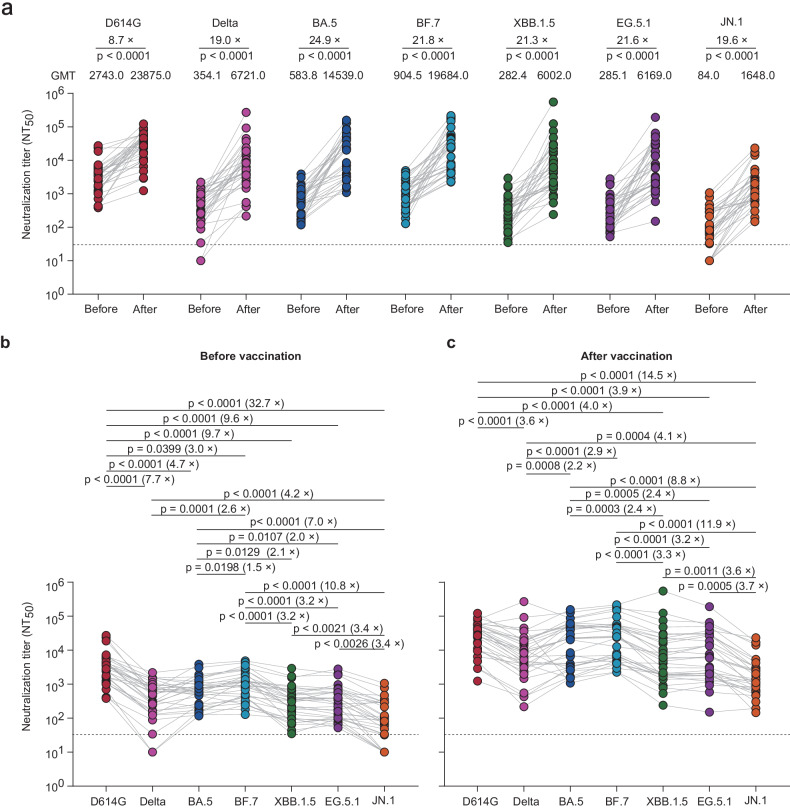
Neutralizing antibody titers before and after XBB.1.5 recombinant COVID-19 trivalent protein vaccine vaccination. **a** Neutralization of pseudoviruses bearing the spike proteins of D614G, Delta, BA.5, BF.7, XBB.1.5, EG.5.1, and JN.1 by individual-matched serum (n = 32) obtained before or after vaccination with the trivalent XBB.1.5 vaccine. Sera were collected from 32 individuals who had experienced a BA.5/BF.7 breakthrough infection in December 2022 and subsequent reinfection (s) of XBB subvariants in 2023 before receiving trivalent XBB.1.5 vaccination. Sera were collected before vaccination (“before”) and three weeks after trivalent XBB.1.5 vaccination (“after”). Each dot represents the 50% neutralization titer (NT_50_) for an individual, and a line connects the NT_50_ values for the same individual before and after vaccination. The geometric mean titer (GMT) and the fold change after vaccination are indicated above the graphs. Statistically significant differences in neutralizing antibody titers before and after vaccination were assessed using a two-sided Wilcoxon signed-rank test. **b**, **c** The data presented in A were regrouped to compare differences in SARS-CoV-2 lineage-specific neutralization before (**b**) and after (**c**) vaccination. The fold change in the GMT is denoted in brackets. A two-tailed Friedman test with a false discovery rate for multiple comparisons was performed. The horizontal dotted line in all the graphs represents a limit of detection of 30, and serum samples with neutralization of less than 30 were plotted as 10. The data for each individual was from a single experiment with two technical replicates

Our findings demonstrate that the trivalent XBB.1.5 protein vaccine booster dose elicits robust neutralizing antibody responses against previous and contemporary SARS-CoV-2 variants and the new JN.1 variant three weeks after vaccination in individuals who have received four or more vaccine doses and experienced breakthrough infection or reinfection. Several Omicron XBB.1.5-containing vaccines, including monovalent XBB.1.5 mRNA vaccines (BNT162b2, Pfizer-BioNTech; mRNA-1273.815, Moderna) and bivalent mRNA (XBB.1.5 + BA.4/BA.5 mRNA-1273.231, Moderna; XBB.1.5/BQ.1 mRNA vaccine, SYS6006.32, CSPC Pharmaceutical Group) vaccines, as well as trivalent (XBB.1.5/BA.5/Delta, WSK-V102C, WestVac Biopharma) and tetravalent protein vaccines (Beta/BA.1/BQ.1.1/XBB.1, SCTV01E-2, SinoCellTech), have been approved for emergence use. Preliminary analysis and observational studies have shown that these XBB.1.5-containing vaccines, whether monovalent,^1^ bivalent,^5,6^ or multivalent,^7^ elicited potent and diverse neutralizing responses against Omicron XBB-lineage variants, as well as the divergent variants EG.5.1, FL.1.5.1, BA.2.86, HK.3.1, HV.1, and JN.1. Importantly, the monovalent XBB.1.5 booster induced neutralizing antibody titers comparable to those induced by XBB infection,^1^ and the SCTV01E booster consistently generated greater neutralizing titers against XBB and pre-XBB variants than against BA.5/BF.7/XBB breakthrough infection.^7^ Moreover, the monovalent XBB.1.5 mRNA vaccine (mRNA-1273.815, Moderna) induced numerically greater neutralizing antibodies than the bivalent XBB.1.5 mRNA vaccine.^5^ Our results are largely consistent with those for updated XBB.1.5-containing monovalent and bivalent mRNA vaccines and tetravalent protein vaccines that have a robust ability to neutralize variants despite antigenic divergences from XBB.1.5.

It should be noted that the serum samples of the study were collected 21 days after vaccination, and it is plausible that the immune response had matured by this time point. Although the absence of B and T cell response assessments is another limitation, these results suggest that the trivalent XBB.1.5 vaccine will likely enhance protection against COVID-19 caused by currently circulating XBB subvariants and the new JN.1 variant. Our findings indicate increased vaccine-induced protection against both antigenically matched variants and the more distant JN.1 variant, supporting current vaccine strategies recommending a trivalent XBB.1.5 vaccine booster dose for older individuals in China. Nonetheless, further investigations are warranted to assess humoral immune responses to the trivalent XBB.1.5 vaccine in older individuals.

### Supplementary information

Supplementary Appendix

## Data Availability

All data supporting the findings of this study are available in the main text and its supplementary information. Raw data and further information are available from the corresponding authors on request.
