# Supplementary material for: Robust neutralizing antibody response to the XBB.1.5 trivalent recombinant protein vaccine booster
Source: Signal Transduct Target Ther. 2024 Aug 16;9:206. doi: 10.1038/s41392-024-01924-y (PMC11327361; doi:10.1038/s41392-024-01924-y)
Supplement: Supplementary file 1 — Supplementary Appendix [file 41392_2024_1924_MOESM1_ESM.docx]

**Supplemental Appendix**

**Robust neutralizing antibody response to the XBB.1.5 trivalent recombinant protein vaccine booster**

**Contents**

| Supplementary Methods | 2-4 |
| --- | --- |
| Supplementary references | 4 |

**Supplementary Methods**

**Study participants and serum sampling**

We included serum samples from 32 individuals who received a 30 μg Coviccine® Trivalent XBB.1.5-Recombinant COVID-19 Trivalent (XBB.1.5+BA.5+Delta; XBB.1.5, 20 μg; BA.5, 5 μg; and Delta, 5 μg) protein vaccine (WSK-V102C; WestVac Biopharma Co., Ltd., China) booster in December 2023. Before the trivalent XBB.1.5 vaccination, these individuals experienced BA.5/BF.7 breakthrough infections during the BA.5/BF.7 wave in China in late 2022 and subsequently experienced reinfection(s) between May and June 2023. The median age was 29 years, and 62.5% were male. The majority of the individuals received 3-4 doses of inactivated vaccines prior to infection. All of them experienced a breakthrough infection during the BA.5/BF.7 wave in late December 2022, and more than half of them had additional reinfection since the breakthrough infection and before trivalent XBB.1.5 vaccination. Serum samples were collected before and 21 days after booster vaccination. All participants completed an online or paper questionnaire that collected demographic and vaccination information and information about their COVID-19-related medical history. The vaccination records of each participant were confirmed through the vaccination system. Individuals with breakthrough infection or reinfection were identified through PCR or self-testing for COVID-19 using a rapid diagnostic test for the SARS-CoV-2 antigen. Informed written consent was obtained from all participants.

**Cell lines**

Human embryonic kidney HEK-293T cells were cultured at 37°C and 5% CO2 in Dulbecco’s modified Eagle’s medium (DMEM, Gibco) supplemented with 10% (v/v) heat-inactivated fetal bovine serum (FBS, Gibco) and supplemented with 1% penicillin–streptomycin (Gibco). The cells were disrupted at confluence with 0.25% trypsin in 1 mM EDTA (Solarbio) every 48–72 h. HEK-293T-hACE2 cells were cultured under the same conditions.

**Spike plasmid pseudovirus production**

Pseudovirus particles were generated as previously described1-6 by cotransfecting HEK-293T cells (ATCC, CRL-3216) with human immunodeficiency virus backbones expressing firefly luciferase (pNL4-3-R-E-luciferase) and the pcDNA3.1 vector encoding the S protein of the D614G, Delta, BA.5, BF.7, XBB.1.5, EG.5.1, and JN.1 plasmids. Codon-optimized, full-length open reading frames of the spike genes of the D614G, Delta, BA.5, BF.7, XBB.1.5, EG.5.1, and JN.1 strains were synthesized by GenScript (Nanjing, China). All plasmid spike sequences were verified by Sanger sequencing. Pseudovirus particles were generated by cotransfecting HEK-293T cells (ATCC) with human immunodeficiency virus backbones expressing firefly luciferase (pNL4-3-R-E-luciferase) and the pcDNA3.1 vector encoding the S protein D614G, Delta, BA.5, BF.7, XBB.1.5, EG.5.1, and JN.1 plasmids. The medium was replaced with fresh medium at 24 h, and the supernatants were harvested at 48 h post-transfection and clarified by centrifugation at 300 × *g* for 10 min before being aliquoted and stored at -80°C until use.

**Pseudovirus neutralization assay**

A SARS-CoV-2 pseudovirus neutralization assay (pVNT) was performed as previously described^1-6^ with the target cell line 293T overexpressing hACE2 orthologs. All viruses were first titrated to normalize the viral input between assays. Duplicate 3-fold 6-point serial dilutions of heat-inactivated sera (spanning 1:30 to 1:7290) were incubated with 500-1000 TCID_50_ of the SARS-CoV-2 pseudotyped virus for 1 h at 37°C and 5% CO_2_. Subsequently, 1x10^4^ 293T-ACE2 cells were added to each well and incubated at 37°C and 5% CO_2_ for 48 h. Afterward, the supernatant was removed, and the cells were lysed using passive lysis buffer (Vazyme) for 3 minutes at room temperature. The lysates were transferred to an opaque white 96-well plate, reconstituted luciferase assay buffer (Vazyme) was added, and the proteins were mixed with each lysate. Luminescence was measured immediately after mixing using a GloMax 96 Microplate Luminometer (Promega). The neutralization titer (NT_50_) was determined by luciferase activity with a four-parameter nonlinear regression inhibitor curve in GraphPad Prism 9.0.0 (GraphPad Software). The NT_50_ was reported as the reciprocal serum dilution causing a 50% reduction in relative light units. If a serum sample had an antibody titer >7290, and it was further serially diluted to determine precise antibody titer. A sample with an NT_50_ value no more than 30 (the detectable limit) was considered to be negative for neutralizing antibodies and was assigned a nominal value of 10 in geometric mean titer (GMT) calculations, which is the lowest serum dilution factor used in the pseudovirus neutralization assay.

**Statistical analysis**

We performed a descriptive analysis summarizing the demographics of the participants and relative frequencies if categorical and with medians and interquartile ranges if continuous. A Wilcoxon matched-pairs signed rank test was used to compare the neutralizing antibody titer before and after vaccination. The Friedman and Kruskal‒Wallis tests with the false discovery rate method were used for multiple comparisons where needed. All the statistical analyses were performed using GraphPad Prism (version 9.0.0, La Jolla, California, USA), and all the statistical tests were 2-sided with a significance level of 0.05.

**Supplementary references**

1 Zhu, K. L. et al*.* Durability of neutralization against Omicron subvariants after vaccination and breakthrough infection. *Cell reports* **42**, 112075 (2023).

2 Wang, X. J. et al*.* Neutralization sensitivity, fusogenicity, and infectivity of Omicron subvariants. *Genome medicine* **14**, 146 (2022).

3 Duan, L. J. et al*.* Neutralizing immunity against SARS-CoV-2 Omicron BA.1 by infection and vaccination. *iScience* **25**, 104886 (2022).

4 Jiang, X. L. et al*.* Omicron BQ.1 and BQ.1.1 escape neutralisation by omicron subvariant breakthrough infection. *Lancet Infect Dis* **23**, 28-30 (2023).

5 Yao, L. et al*.* Omicron subvariants escape antibodies elicited by vaccination and BA.2.2 infection. *Lancet Infect Dis* **22**, 1116-1117 (2022).

6 Duan, L. J. et al*.* SARS-CoV-2 vaccine-induced antibody and T cell response in SARS-CoV-1 survivors. *Cell reports* **40**, 111284 (2022).
